# Characterization of Astrocytic Response after Experiencing Cavitation In Vitro

**DOI:** 10.1002/gch2.201900014

**Published:** 2020-05-17

**Authors:** Alex H. Wrede, Marilyn C. McNamara, Rodger Baldwin, Jie Luo, Reza Montazami, Anumantha Kanthasamy, Nicole N. Hashemi

**Affiliations:** ^1^ Department of Mechanical Engineering Iowa State University Ames IA 50011 USA; ^2^ Department of Biomedical Sciences Iowa State University Ames IA 50011 USA

**Keywords:** astrocytes, cavitation, microbubbles, traumatic brain injuries, ultrasonics

## Abstract

When a traumatic brain injury (TBI) occurs, low‐pressure regions inside the skull can cause vapor contents in the cerebral spinal fluid (CSF) to expand and collapse, a phenomenon known as cavitation. When these microbubbles (MBs) collapse, shock waves are radiated outward and are known to damage surrounding materials in other applications, like the steel foundation of boat propellers, so it is alarming to realize the damage that cavitation inflicts on vulnerable brain tissue. Using cell‐laden microfibers, the longitudinal morphological response that mouse astrocytes have to surrounding cavitation in vitro is visually analyzed. Astrocytic damage is evident immediately after cavitation when compared to a control sample, as their processes retract. Forty‐eight hours later, the astrocytes appeared to spread across the fibers, as normal. This study also analyzes the gene expression changes that occur post‐cavitation via quantitative polymerase chain reaction (qPCR) methods. After cavitation a number of pro‐inflammatory genes are upregulated, including TNFα, IL‐1β, C1q, Serping1, NOS1, IL‐6, and JMJD3. Taken together, these results confirm that surrounding cavitation is detrimental to astrocytic function, and yield opportunities to further the understanding of how protective headgear can minimize or eliminate the occurrence of cavitation.

## Graphical abstract

This study demonstrates biocompatibility in an apparatus that engineers controlled cavitation. Visual analytics show that astrocytes undergo significant morphological changes after exposure to cavitation. Genetic analytics are conducted post‐cavitation to show drastic upregulation in classic traumatic brain injury–related genes. Astrocytes are known to be neurotoxic after cranial insult, and this study confirms the adverse effects that cavitation has on the astrocytic phenotype.

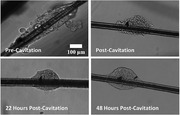

## 1 Introduction

Traumatic brain injuries (TBIs) are a large problem in modern society. Estimates show that by the year 2020, TBIs will become the third most prevalent cause of death worldwide.^[^ ^1^ ^]^ TBIs are also a leading cause of death and disability in children and young adults in United States.^[^ ^2^ ^]^ TBIs have two different stages of injury: primary and secondary. A primary injury occurs at the time of the injury, which might be a skull fracture, force‐related stress from the brain shifting within the skull boundary, or cavitation, which is the formation and dramatic collapse of MBs. A secondary injury occurs sometime after the injury, such as intracranial inflammation or blood–brain barrier damage.^[^ ^3^, ^4^ ^]^ This study aims to specifically focus on the damages that the primary injury of cavitation has on surrounding neuronal cells and seeks to elucidate the unknown nature and unpredictability of TBIs.

The existence of cavitation inside the skull during a TBI situation is a modern hypothesis in research. Goeller et al. used a brain‐like ellipsoid model in a shock tube to confirm the existence of cavitation in a TBI environment.^[^ ^5^ ^]^ During a blast‐TBI, the initial compressive wave travels through the skull and a fraction of this wave reflects off impedance boundaries, resulting in a negative pressure reflection wave.^[^ ^6^ ^]^ It is in these low pressure regions where intracranial cavitation exists.^[^ ^7^, ^8^ ^]^ The CSF has minute pockets of vapor because of its primary components, which are oxygen, nitrogen, and water vapor.^[^ ^9^ ^]^ When these vapor pockets are exposed to low pressure conditions they dramatically expand and collapse when the bubble radius exits the stability region.^[^ ^10^ ^]^ This expansion and collapse creates an intense localized force measured anywhere from 0.1–20 MPa.^[^ ^11^ ^]^ Cavitation damage has been heavily investigated in mechanical pump and watercraft propulsion applications.^[^ ^12^, ^13^, ^14^ ^]^ Characterization of the impact that cavitation has inside the skull during a TBI remains to be fully explored, and thus is the ultimate novelty of this study.^[^ ^15^ ^]^

On top of TBIs, there are other profound neurological disorders, such as, Alzheimer's disease, Parkinson's disease, and psychiatric diseases, affecting large portions of the population. Research has shown a pro‐inflammatory response in the pathogenesis during these situations.^[^ ^16^, ^17^ ^]^ Astrocyte activation plays a vital role in initiating neuroinflammation.^[^ ^18^, ^19^ ^]^ Astrocytes are bountiful in the CNS and provide a variety of functions in the neuronal network, such as, supplying support and structure to neurons, and enhancing synapse formation and function.^[^ ^20^ ^]^ Upon insult, though, the full functionality of astrocytes may be lost. Liddelow et al. categorized astrocyte reactivity into two groupings, called A1 and A2 astrocytes.^[^ ^19^ ^]^ A1 astrocytes are the result of an inflammatory response similar to what is observed during a blast‐TBI, and are known to be harmful because they lose regular astrocytic functionality and instead provide a neurotoxic function that kills neurons. A1 astrocytes upregulate many genes that are known to be damaging to synapses. However, A2 astrocytes, which are activated through ischemia, are shown to be protective by further promoting CNS repair and recovery.^[^ ^21^, ^22^, ^23^ ^]^ We postulate that cavitation will induce astrocyte cells toward the A1 astrocyte phenotype and upregulate mostly A1 specific genes, also modeled by Liddelow et al.^[^ ^19^ ^]^ Because of the large role that astrocyte cells play in TBI response, this study is solely focused on the investigation of astrocyte activation in response to surrounding cavitation. Future studies involving cocultures of multiple types of neuronal cells will further elucidate this phenomenon by lending further physiological relevancy.

Neuronal cells are also known to retract their processes during a blast‐TBI.^[^ ^24^ ^]^ The model analyzed in our study demonstrates this retraction function in astrocytes by conducting a time‐dependent visual study of cells at different stages post cavitation. We compare this collage with a control sample to demonstrate that cavitation is a primary inducer for astrocytes to portray an amoeboid morphology. These results motivated a genetic study to further investigate the A1 versus A2 expressions in astrocytes post‐cavitation. Our hypothesis is that the astrocytes that experience damage from surrounding cavitation will represent the phenotype of A1 astrocytes because this is an inflammatory insult. Some classic genes that show upregulation in pro‐inflammatory astrocytes include, Tumor Necrosis Factor alpha (TNFα), Interleukin 1‐beta (IL‐1β), C1q, Serping1, and other reactive oxygen species.^[^ ^25^, ^26^, ^27^, ^28^ ^]^ Related studies conclude that unknown oxygen species are secreted by astrocytes and in turn, act as neurotoxins.^[^ ^29^ ^]^ Nitric oxide (NO), more specifically neuronal nitric oxide synthase (NOS1), plays a vital role in the overall function of the CNS.^[^ ^30^ ^]^ In neurodegenerative diseases like Parkinson's disease, stroke, Alzheimer's disease, and amyotrophic lateral sclerosis, there has been evidence of increased NOS1 expression.^[^ ^30^, ^31^, ^32^, ^33^, ^34^ ^]^ It has been proven that increasing concentrations of TNFα and IL‐1β also increases the presence of interleukin 6 (IL‐6) in mouse astrocytes.^[^ ^24^ ^]^ This trend is magnified in human astrocytes.^[^ ^35^ ^]^ IL‐6 has also been associated with multiple sclerosis (MS).^[^ ^36^ ^]^ An increased concentration of jumonji domain containing 3 (JMJD3) also has been proven to lead to upregulation in IL‐6.^[^ ^37^ ^]^ On top of this, increasing levels of JMJD3 has been shown to activate the expression of a variety of pro‐inflammatory genes in neuronal cultures.^[^ ^18^, ^37^ ^]^ This study focuses on characterizing the expression of TNFα, IL‐1β, C1q, Serping1, NOS1, IL‐6, and JMJD3 in mouse astrocytes cells after experiencing surrounding cavitation. We also wanted to analyze some anti‐inflammatory (A2 phenotype) genes to see if cavitation solely had influence on A1 specific genes. The well‐known anti‐inflammatory genes that we test are tm4sf1, sphk1, CD14, IL13, and Arginase1.^[^ ^19^, ^26^, ^28^, ^38^ ^]^ For all of our gene expression studies we used qPCR methods. A detailed summary of these gene expression changes has the potential to unmask important specifics about the nature of TBI at the cellular level.

Previous studies have designed an apparatus that creates controlled cavitation. This process produces, traps, positions, and collapses MBs arbitrarily in a tank using microfiber adhesion and an ultrasound transducer. This apparatus was also designed to realistically mimic the cavitation phenomena that exists during a TBI.^[^ ^39^ ^]^ Our study seeks to advance this design by making it biocompatible and sterile for cell incorporation. Introducing astrocytes in this apparatus and performing visual and gene expression analytic techniques, we demonstrate that cavitation has an adverse effect on astrocytic morphology and genetics.

## 2 Results and Discussion

### 2.1 Visual Morphology Analysis

**Figure 1:**
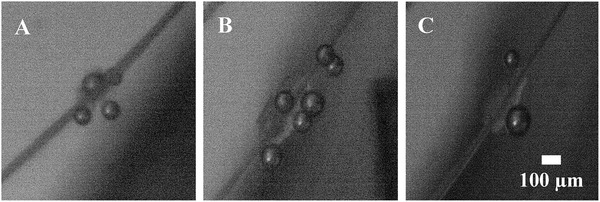
A–C) Illustration of some different arrangements of MBs adhered to healthy clusters of astrocyte cells during experimentation. Higher magnification of cell morphology via an inverted microscope is shown in Figure 3. These images are taken with the samples inside the PBS tank.

**Figure 2:**
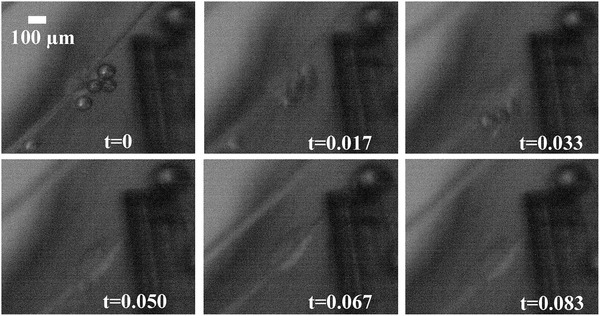
Longitudinal collage showing cavitation on cell‐laden microfibers. The samples are in the PBS tank and pictures are taken with high‐speed camera and telescopic lens. At *t* = 0 the ultrasonic transducer is activated. The later frames show the dramatic oscillation and collapse of MBs in response to ultrasound exposure.

With our advanced apparatus, it is possible to achieve a wide range of control over the configuration of the microfiber‐cell‐MB complex. **Figure** **1** illustrates typical arrangements of trapped MBs next to clusters of cells in the apparatus. We have arbitrary control where the MBs adhere, the number of MBs that adhere, and the size of the MBs, because the microfiber scaffold is attached to a 3‐axis stage and easily adjusted to enter the plane of the rising MBs, allowing for MBs to coalesce allows for varying MB sizes. In Figure 1C, a 60 µm bubble is adhered to the cells on the top of the cell group and a larger MB around 100 microns is placed on the bottom of the grouping. The 100 µm bubble was created by allowing two 60 µm bubbles to coalesce. After an arbitrary amount of MBs are collected on the cell‐laden microfibers, the ultrasonic transducer is activated, resulting in cavitation. **Figure** **2** shows a collage that illustrates live cavitation on cell‐laden microfibers.

**Figure 3:**
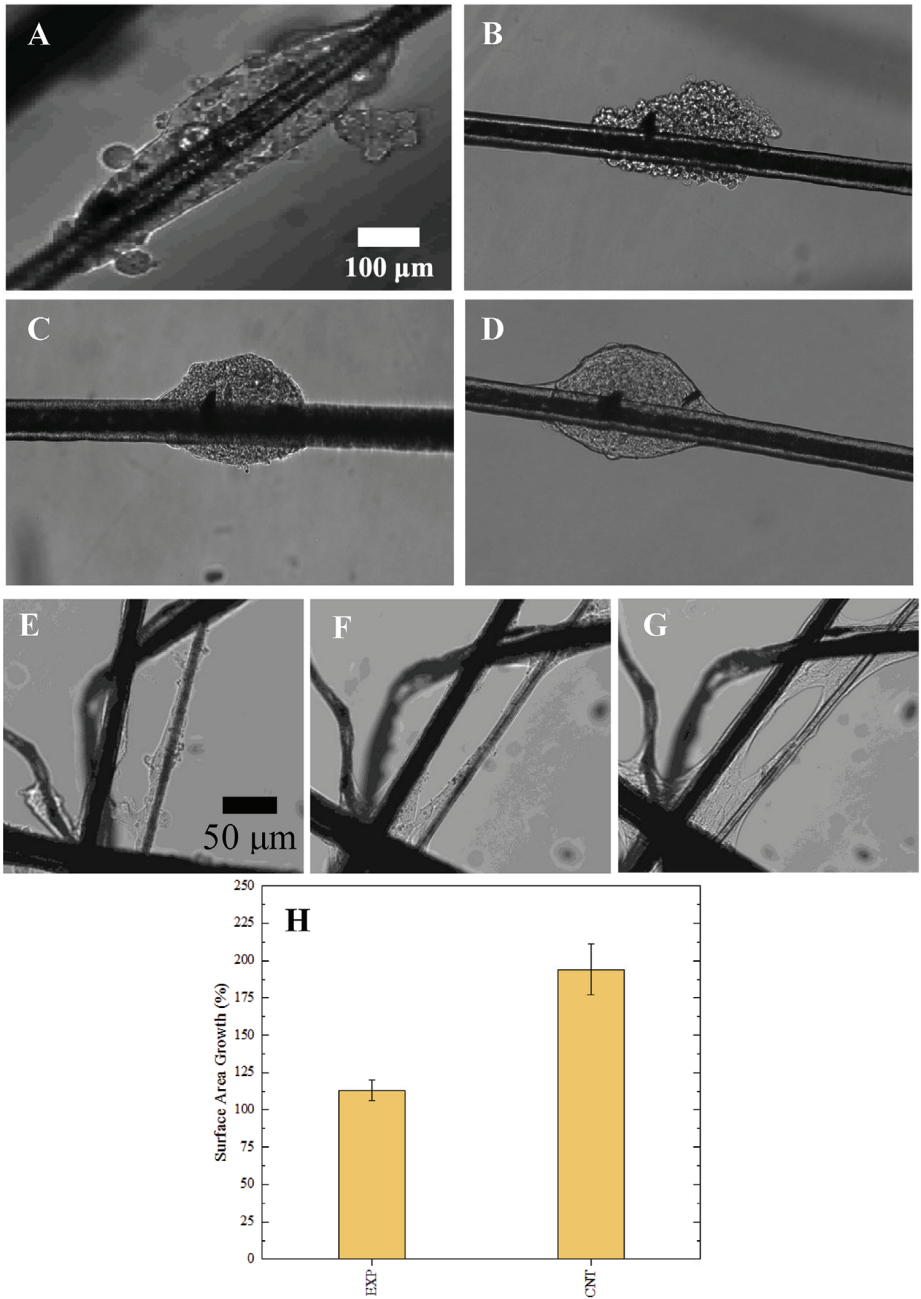
A–D) Visual analysis of mouse astrocyte response the cavitation. These images are captured via an inverted microscope outside of the PBS tank and the sample is in a cell culture plate. A) Astrocyte sample before experiencing PBS, ultrasound, and cavitation exposure. After they are exposed, we visualize their response immediately B) after treatment, C) 22 h after treatment, and D) 48 h after treatment. A–D) Scale 100 microns. E–G) Visual analysis of astrocytic response to PBS and ultrasound exposure, but no exposure to cavitation. This sample is used as a control. E) Represents the sample immediately after exposure, F) represents the sample 22 h after exposure, and G) represents the sample 48 after exposure. E–G) Scale 50 microns. H) Growth analysis 48 h after control and experimental exposures. Magnitudes are percentages relative to the area measured immediately after experiencing control or experimental conditions. Error bars represent ±1 standard deviation.

The astrocytic response is illustrated through a comparative analysis with a control and experimental sample in order to justify that cavitation is the leading contributor to inducing reactive astrocytes. The control sample was submerged in the PBS tank and also experiences ultrasound exposure from the transducer. There are not any adhered MBs on the control sample so cavitation does not exist when the transducer is activated. The experimental sample is exposed to PBS, ultrasound exposure, and surrounding cavitation because of the presence of adhered MBs prior to activating the ultrasonic transducer. **Figure** **3** shows the longitudinal astrocytic response for the experimental (A–D) and control samples (E–G), respectively. In Figure 3A–D, this particular sample had five ≈60 µm MBs adhered to the group of cells prior to activating the ultrasonic transducer. A growth analysis was done to quantify the retraction of processes and total surface area in both experimental and control samples. The results concluded that both samples increased in surface area 48 h after control and experimental exposures, but the magnitude of growth was significantly different. The control sample (Figure 3E–G) grew at a rate of 200% and the experimental sample (Figure 3A–D) grew 109%, both over 48 h. Figure 3H illustrates the quantitative astrocytic surface area growth in all of our trials.

### 2.2 Genetic Analysis via qPCR

**Figure 4:**
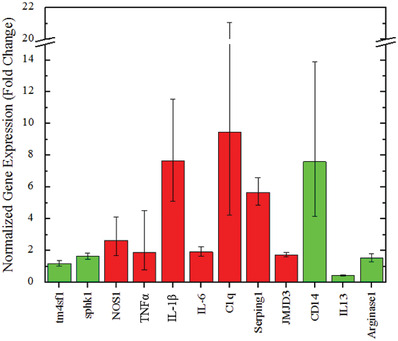
Gene expression analysis of mouse astrocytes immediately after exposure to surrounding cavitation in the PBS tank. Expressions are represented as a fold change between the control sample and treatment sample of each targeted gene. Genes labeled with red bars designate A1 specific genes and genes labeled with green bars designate A2 specific genes.

Through our visual analysis studies we were able to see that astrocytes retract their processes immediately after exposure to cavitation and they begin to elongate overtime. We further investigate this response by studying the A1 and A2 genetic phenotype immediately after cavitation. This is done via qPCR methods and using the cDNA from the separated control and experimental samples. **Figure** **4** outlines the astrocyte phenotype represented by astrocytes that have been activated via cavitation. The A1 specific genes that we test are: TNFα, IL‐1β, C1q, Serping1, NOS1, IL‐6, and JMJD3. The A2 specific genes that we test are: tm4sf1, sphk1, CD14, IL13, and Arginase1. Expression values are represented as normalized fold changes from the control and treatment samples from each of these genes.

The presence of cavitation inside the skull during a TBI is a modern hypothesis. Some studies have recently proven the existence of this phenomenon, but there is limited knowledge on the characterization of cellular response in this scenario.^[^ ^5^, ^40^ ^]^ This study used controlled cavitation techniques from previous studies and made modifications and advancements in order to obtain biocompatibility and sterility, allowing for the incorporation of mouse astrocytes.^[^ ^39^ ^]^ We used astrocytes in this study because of their documented role in TBI response and recovery.^[^ ^19^, ^29^ ^]^

In future studies, we plan to further characterize the radial response that astrocytes undergo post‐cavitation via advanced immunocytochemistry techniques. Localizing the cavitation at the center of the coverslips and analyzing the radial morphology changes will be the centralized intention of this study. Future studies also aim to incorporate multiple types of neuronal cells in order to further characterize the damages that cranial cavitation has on surrounding anatomy.

One of the novelties of this study is ability to visualize the cellular response of a finite amount of cells by using cell‐laden microfibers. In the visual analysis we were able to see morphology differences between control and experimental samples. In the experimental sample the astrocytes clearly retract their processes and represent an amoeboid morphology. This is shown in Figure 3B where the cell grouping has a very rough surface. In the control sample there is also some evident cellular disruption (Figure 3E) but the cells appear displaced, instead of damaged. The outer boundaries of the cell groupings do not have this rough nature, like the experimental sample. The displacement of cells in the control sample is likely due to the transportation of the sample from the PBS tank to cell culture plate. After 22 and 48 h both samples appear to expand their processes and spread across the fibers. For both experimental and control samples the total measured surface area increases 48 h after relative exposures, however, the control samples grow significantly faster compared to the experimental samples. This dramatic increase in surface area for the control samples is likely due to the astrocytic function of forming protective scar tissue when entering the PBS tank.^[^ ^41^ ^]^ A1 astrocytes lose this functionality and no longer protect surrounding neurons.^[^ ^19^ ^]^

Given the difference in morphology immediately after experimental and control exposures, we sought to investigate the genetic differences at this time point. Future studies aim to characterize the genetic tendencies longitudinally, for it is known that genetic phenotypes change overtime.^[^ ^41^ ^]^ Current technology is limited in offering analytic techniques to gather genetic phenotypes of small amounts of cells, like the cell groupings on microfibers. Techniques like qPCR require large amounts of cells due to sensitivity constraints. Single‐cell RNA sequencing is an evolving method that offers this ability but we have limited access and resources to pursue this technique. We instead engineered our apparatus to allow for greater amounts of cells and the incorporation of qPCR. We did this by replacing the cell‐laden microfiber scaffold with sterilized cell‐seeded coverslips. Due to the increase in cell amount and sample area, we also increased the number of adhered MBs to 30–40 per sample instead of 5–10, like what was adhered to the cell‐laden microfibers. The gene expression results show a clear difference between experimental and control phenotypes. All selected A1 markers were unregulated after cavitation. The following genes also had at least +2 fold change: IL‐1β, C1q, Serping1, and NOS1, analogous to literature.^[^ ^19^, ^29^, ^30^ ^]^ Of the A2 markers that were tested, the only A2 gene that was unregulated with a fold change of 2+ was CD14. These A1 and A2 genetic findings lead us to conclude that surrounding cavitation induces A1 reactive astrocytes and in turn, can be detrimental to the neuronal network. It is aforementioned that NOS1 expression is directly correlated to the presence of neurodegenerative diseases.^[^ ^30^, ^31^, ^32^, ^33^, ^34^ ^]^ Increased levels TNFα, IL‐1β, and JMJD3 spike the levels of IL‐6 and, in turn, has been directly associated with MS.^[^ ^24^, ^36^, ^37^ ^]^ It is noticed that there is some large variances for some of the genes in Figure 4. We were not surprised by this because of the nature of cavitation and the varied damage distribution across the coverslip surface. In our methodology a finite amount of MBs are captured on the coverslip surface and in return, the cavitation force localized to these regions. ^[^ ^42^ ^]^ Astrocytes that are not nearby these cavitation regions experience dampened exposures from cavitation and reduced overall damage. Reduced damage exposure also leads to varied gene expressions levels, analogous to the increased variation in our results. The specific magnitude of gene expression changes are difficult to determine from our results. The main objective of this study, though, is to analyze individual gene expression trends following cavitation exposure. More specifically, the low bounds of the standard error bars still show upregulation for the A1 specific genes, giving a clear conclusion in gene trends. The variance found in the results adds additional novelty instead of statistical insignificance, because despite the methodological efforts to centralize the cells on the coverslips, the astrocytes still represented different phenotypes bases on the degree of cavitation exposure. These results help further understand this unique phenomena that cavitation has during a TBI.

Due to the upregulation of CD14 we conclude that cavitation induces a unique astrocytic phenotype that is similar to A1 inducing injuries, but not entirely similar. We postulate that the slight differences in A1 versus A2 consistency is due to the unique damage that cavitation leaves behind‐ cavitation is a very localized force that often collapses on the microsecond scale. We conclude our results by introducing A3 reactive astrocytes‐ astrocytes that are highly similar to A1 astrocytes but have slight differences due to the uniqueness of the damage exposure (e.g., cavitation).

Our genetic findings are significant because if cavitation is proven to be a leading contributor in A1 induction then this provides another avenue in preventative care. The pharmaceutical attention goes toward curing the damage that A1 astrocytes endow, but the engineering attention can now have a focus in studying where cavitation specifically occurs in the brain. A location map of cavitation occurrence can be directly used to design a helmet that dampens force and reduces the possibility of cavitation, which in turn, reduces neurotoxicity in astrocytic response and minimizes TBI detriments. Additional genes should also be studied in the future to see if cavitation promotes the upregulation of additional A2 genes. If more A2 genes are upregulated then the A3 phenotype that cavitation induces may be less harmful to the neuronal network compared to classic A1 astrocytes.

## 3 Conclusion

TBIs are complex injuries that often times lead to unpredictable symptoms for its victims. There are primary and secondary injuries that occur during a TBI. Breaking down these injuries and studying the effects that they endow on the cellular levels is important in order to gain knowledge on the topic. An example of a primary injury is cavitation that exists in low pressure regions of the brain. We made advancements to an apparatus designed in previous studies that creates, traps, positions, and collapses MBs that realistically resemble those in a blast‐TBI event.^[^ ^39^ ^]^ Our study uses PCL microfibers to mimic a biocompatible and sterile neuronal environment for mouse astrocyte cells in‐vitro. We document a visual study that demonstrates surrounding cavitation has a profound effect on astrocytic activation. We also use qPCR techniques to investigate the effects that surrounding cavitation has on the genetic phenotype of astrocytes. This study has proven that astrocytic function drastically changes upon exposure to surrounding cavitation. A1 genetic tendencies are consistently represented and known to be neurotoxic. Cavitation is found to induce a unique reactive astrocyte type, which we have termed as A3. This study can be used as a platform in efforts to eliminate cavitation in the brain through advanced helmet design. Further research focusing on the response of multiple types of neuronal cells after experiencing surrounding cavitation will be vital in continuing the advancement to TBI prevention and care.

## 4 Experimental Section

##### Biocompatibility and Sterilization

Previous studies describe the apparatus used to create controlled cavitation by having the ability to arbitrarily produce, trap, position, and collapse MBs.^[^ ^39^ ^]^ In order to make this apparatus sterile and stable for live cellular testing there are multiple advancements that were implemented. Traditionally cellular testing is done underneath a sterilized fume hood but with the necessary components for MB creation, positioning, and collapse, conducting experiments under a fume hood for this project is impractical. The 3‐axis stages and high‐speed camera are mounted onto an optics table in order to remain stationary, which a biological fume hood environment is unable to provide. In order to obtain sterility and stability for cellular analysis a number of advancements were made. The 1.5 gallon tank was filled with 3.5 L of phosphate buffered saline (PBS) instead of deionized water to account for cell compatibility. Before the PBS was put into the tank, the tank was first sprayed down with 70% ethanol and put in a sterilized fume hood until dry under UV. Then the PBS was poured into the tank under the fume hood and exposed to 45 min of UV light for further sterilization. Also, the pieces of the mechanical arm that was submerged in the PBS and used to hold the polycaprolactone (PCL) frame stationary between the capillary tubing and transducer were also sterilized with 70% ethanol and exposed to UV light for 45 min. The surrounding shelves and walls were also draped with sterilized plastic to eliminate any dust or debris falling into the tank during experimentation. Additionally, all other surrounding components (capillary tubing, 3‐axis stage, transducer and the mechanical arm that held it stationary, and the transducer cords) were sprayed and wiped with 70% ethanol continuously prior to the introduction of cells. After the PBS filled tank was sterilized, it was then covered with a sterile sheet and placed in an oven to heat at 70 °C for 1 h. This allowed for the PBS to warm to about body temperature so the cells would not experience a temperature shock when being introduced in the tank. The cell‐laden fiber frames were kept in a sterile and stable incubator before and after trials. Using these advancements, it was possible to eliminate any signs of infection or contamination in all of the samples.

##### Fibrous Frame Fabrication

Frames were fabricated by encapsulating copper within Polydimethylsiloxane (PDMS) (1:10 Base:Elastomer) in a “U” shape, since exposed copper caused cell death and PDMS floated within the media and exposed fibers to the air. Fibers were fabricated using a well‐documented protocol involving a microfluidic device.^[^ ^43^, ^44^ ^]^ Specifically, 5% PCL (*M*_n_ = 80 000, Sigma‐Aldrich, St. Louis, MO) was dissolved into 2,2,2‐Trifluoroethanol (TFE, Oakwood Chemical, West Columbia, SC) to form the core solution, while 5% Polyethylene glycol (*M*_n_ = 20 000, Sigma‐Aldrich, St. Louis, MO) was dissolved into a 1:1 solution of DI water and ethanol for a sheath fluid. Fibers were the result of a 5:75 µL min^−1^: µL min^−1^ flow rate ratio. Fibers were wrapped around the aforementioned frames in a ratio of 10 fibers per frame.

##### Cell Model

The mouse astrocytes (CRL‐2541) used in this project were purchased from American Type Culture Collection (ATCC, Manassas, VA) because of their known utility in TBI studies.^[^ ^45^ ^]^ These cells are specifically clonal permanent cell lines with astrocytic properties, instead of being solely an astrocyte culture. This culture provides a solid foundation for the response that neuronal cells have to cavitation and future studies aim to use primary astrocyte cultures. The cells were grown according to company protocol in growth media (ATCC, 30‐2002). The culture media was also supplemented with 10% FBS and 1% penicillin (10 000 U mL^−1^)‐ streptomycin (10 000 µg mL^−1^), both of which were purchased from Gibco (Waltham, MA). The cells in all of the data collection consist of culture passage numbers 3–7. The astrocytes were housed in an incubator set at 37 °C with 5% CO_2_.

##### Visual Morphology Analysis

Cells were seeded onto biocompatible PCL microfibers (Hashemi Lab, Iowa State University, Ames, IA) to promote cell adhesion and allow for groupings of finite amounts of cells.^[^ ^46^, ^47^ ^]^ The cells were suspended at a density of 1.06 × 10^6^ cells mL^−1^ before 0.25 mL of cells were introduced to the fibers and cultured overnight prior to running experiments. A large fraction of the seeded cells settled on the bottom of the culture flask but some adhered to the microfibers. The apparatus layout for the technique was similar to that of the microfiber adhesion technique documented in previous studies.^[^ ^39^ ^]^ The function generator input settings for the ultrasonic cavitation induction were: 5 cycles, pulse repetition frequency 59 Hz, 130 Vpp. The culture tank is filled with PBS and has a measured pH value of 7.35. Using the microfiber adhesion method there is arbitrary control over the number of MBs that are captured on the cell‐laden microfibers. All images of MBs adhered to cell‐laden microfibers are captured using a long‐distance microscope (Model K2, Infinity Photo‐Optical Company) in unison with a high speed camera for imaging (BlackFly, FLIR) while the samples are in the 1.5 gallon tank. After cavitation, the samples were individually placed in 12‐well plates with fresh cell culture media and all images are captured using an inverted microscope (Zeiss, Germany). The post‐cavitation images are collected at timepoints from 0–48 h. A growth analysis was conducted in order to quantify the changes that are observed between the experimental and control samples. The total area of growth in the plane of the inverted microscope was quantified and measured using imageJ software (Wayne Rasband, NIH).

##### Gene Expression Analysis via qPCR

**Figure 5:**
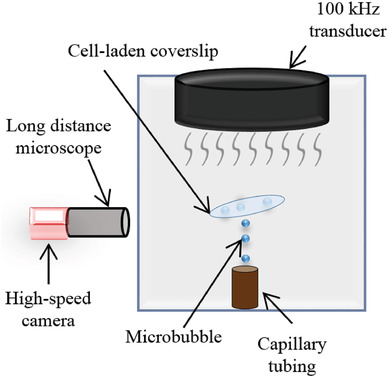
Apparatus configuration for genetic analysis. Coverslips are suspended using a mechanical arm that is stationed on a 3‐axis stage, allowing for capture of MBs at arbitrary positions on the coverslip. When the coverslip has an optimal amount of adhered MBs on its underside, it is rotated 180° so the MBs and cells are exposed to the ultrasound.

The apparatus configuration of this experiment is similar to that of the visual analytic techniques with one slight modification, the cells are seeded onto sterilized 12 mm coverslips instead of using microfiber scaffolds, shown in **Figure** **5**. There were 40 total seeded coverslips, 20 designated for a control sample and 20 designated for experimental sample. The control and experimental samples each had 1.17 × 10^6^ cells distributed across the 20 coverslips and cultured overnight in the incubator prior to experimentation. During cell seeding procedure, the cells were focused at the center of the coverslip to localize the culture and, in turn, minimized the variance in cavitation that the cells experienced. The cell‐laden coverslips were suspended mid‐solution with a tweezers clamped in a mechanical arm. Control samples were submerged in the PBS tank and exposed to ultrasound (5 cycles, pulse repetition frequency 59 Hz, 130 Vpp), but there were not any MBs present to produce cavitation. The experimental samples experienced an identical environment with the addition of 30–40 MBs adhered on each coverslip prior to activating the ultrasound. After the MBs are collected along the cell‐seeded surface, the coverslips are then manually rotated 180° so the cells and MBs are aligned with beam of the transducer and cavitation is able to occur. After treatments the cell‐laden coverslips were trypsinized, collected, combined to create one control and one treatment sample, pelleted, and frozen at −80 °C until homogenization. The PBS surrounding the coverslips was also centrifuged and combined with the trypsinized cells because some of the astrocytes detached from the coverslips following cavitation. Enhanced cell adhesion to the coverslips would have been possible through using a substrate, but previous studies found that the presence of a substrate can alter the genetic response that a cell culture undergoes.^[^ ^48^ ^]^ Because of this reason it was decided to not coat the coverslips with a substrate. The samples were homogenized in TRIzol reagent (Invitrogen Cat number: 15 596 026). RNA isolation was done following the TRIzol reagent manufacturer's protocol. Next, a cDNA synthesis system, High Capacity cDNA Reverse Transcription Kit, was used to complete a reverse transcription and convert the RNA to cDNA (Applied Biosystems, Foster City, CA). The relative magnitude of gene expression was measured using real‐time PCR with Qiagen RT^2^ SYBR Green master mix and validated Quantitect qPCR mouse primers from Qiagen (Frederick, MD). Mouse gene 18S rRNA (Qiagen Cat. No. PPM57735E) was used as the housekeeping gene, and used in the normalization of each sample. In order to ensure that florescence data from amplicon peaks were not from of any nonspecific amplicons, dissociation and melting curves were ran, according to the protocol from the manufacturer. The final results were calculated using the ΔΔ*C*_t_ method and implementing the threshold cycle (*C*_t_) value for the housekeeping gene and for the respective gene of interest in each sample. The reported expression changes are represented as fold changes between the control sample and treatment sample of each targeted gene.^[^ ^49^ ^]^

## Conflict of Interest

The authors declare no conflict of interest.
